# Fractalkine/CX3CR1 signaling during neuropathic pain

**DOI:** 10.3389/fncel.2014.00121

**Published:** 2014-05-07

**Authors:** Anna K. Clark, Marzia Malcangio

**Affiliations:** Wolfson Centre for Age Related Diseases, King’s College LondonLondon, UK

**Keywords:** microglia, proteases, pain, chronic pain, chemokines

## Abstract

Chronic pain represents a major problem in clinical medicine. Whilst the acute pain that is associated with tissue injury is a protective signal that serves to maintain homeostasis, chronic pain is a debilitating condition that persists long after the inciting stimulus subsides. Chronic neuropathic pain that develops following damage or disease of the nervous system is partially treated by current therapies, leaving scope for new therapies to improve treatment outcome. Peripheral nerve damage is associated with alterations to the sensory neuroaxis that promote maladaptive augmentation of nociceptive transmission. Thus, neuropathic pain patients exhibit exaggerated responses to noxious stimuli, as well as pain caused by stimuli which are normally non-painful. Increased nociceptive input from the periphery triggers physiological plasticity and long lasting transcriptional and post-translational changes in the CNS defined as central sensitization. Nerve injury induces gliosis which contributes to central sensitization and results in enhanced communication between neurons and microglial cells within the dorsal horn. Thus, identification of mechanisms regulating neuro-immune interactions that occur during neuropathic pain may provide future therapeutic targets. Specifically, chemokines and their receptors play a pivotal role in mediating neuro-immune communication which leads to increased nociception. In particular, the chemokine Fractalkine (FKN) and the CX3CR1 receptor have come to light as a key signaling pair during neuropathic pain states.

## Introduction

Acute pain can be regarded as a homeostatic and adaptive process by which the organism becomes aware of harmful stimuli, thus guarding against actual or potential tissue injury. As such, the physiological transduction and transmission of noxious stimuli is a vital protective mechanism (nociceptive pain), allowing withdrawal from potentially damaging environmental factors. Nociceptive pain persists only for the duration of the stimulus or tissue damage. The fundamental importance of pain as a homeostatic mechanism becomes apparent in the case of individuals who have a complete lack of nociception; rare hereditary mutations resulting in congenital insensitivity to pain lead affected individuals to inadvertently inflict injury upon themselves throughout life (Cox et al., 2006).

Under some circumstances pain can outlast its physiological role, developing into chronic pain; a debilitating condition lasting longer than 3 months from the noxious stimulus, during which the pain is out of proportion to the initial inciting injury. Chronic neuropathic pain results from damage to, or dysfunction of, the somatosensory system and is maladaptive in that the pain neither protects the organism nor supports tissue repair. Neuropathic pain is commonly associated with direct trauma (stretch or crush) to a peripheral nerve. In addition, disease states including diabetes mellitus and viral infections may result in neuropathic pain symptoms. Furthermore, pharmacological agents such as anti-retroviral drugs and chemotherapy agents may also result in the development of painful neuropathy following dysfunction of sensory nerves. Neuropathic pain is a complex pain syndrome consisting of multiple symptoms. These include sensory loss, abnormal sensation, spontaneous pain, and alterations in responses to stimulus-evoked pain (hyperalgesia and allodynia) (Jensen et al., 2001; Baron, 2006). Neuropathic pain is a significant clinical problem, for which current treatments are inadequate. This is due in large part to the fact that the mechanisms underlying neuropathic pain syndromes are insufficiently understood.

Convincing pre-clinical evidence suggests that following peripheral nerve injury neuro-immune interactions play pivotal roles in the generation and maintenance of nociceptive hypersensitivity. Cells of the immune system interact with the sensory system at various locations. In the peripheral nerve the infiltration of immune cells (which release both pro-nociceptive and anti-nociceptive mediators) is critical for the early initiation phase of neuropathic pain in rodent models (Austin and Moalem-Taylor, 2010; Stein and Machelska, 2011). In the dorsal horn of the spinal cord disruption of homeostasis and exaggerated primary afferent input causes microglia to transition from surveillance states into pain-related enhanced response states, thus modifying the nature of neuron-microglia communication and promoting a maladaptive augmentation of nociceptive transmission that underlies the chronicity of neuropathic pain.

Neuron-microglia communications in the dorsal horn occur through activation of defined pathways. In particular, two critical neuron-microglia signaling systems initiated by purinergic receptors contribute to nerve injury induced hypersensitivity. A microglia-driven pathway whereby *de novo* P2X4 receptor expression and activation leads to release of Brain-Derived Neurotrophic Factor (BDNF; Ulmann et al., 2008; Trang et al., 2009) is critical during the initiation phase of neuropathic pain (shortly after nerve injury) (Tsuda et al., 2003). BDNF activation of the TrkB receptor down-regulates the expression of the neuronal potassium/chloride co-transporter KCC2 (Coull et al., 2005). The consequential impairment of chloride homeostasis in the superficial laminae of the dorsal horn results in reduced inhibition following GABA_A_ receptor activation (Coull et al., 2005), and therefore a more excitatory environment. The therapeutic exploitability of this P2X4/BDNF/KCC2 pathway is highlighted by the recent identification of chloride extrusion enhancer compounds that exert significant anti-nociceptive effects in neuropathic rats (Gagnon et al., 2013).

We have identified a second neuron-microglia signaling pathway that is critically involved in the maintenance phase of neuropathic pain. This second microglia-driven pathway is initiated by activation of the low affinity P2X7 receptor, resulting in release of the lysosomal protease Cathepsin S (CatS; Clark et al., 2010). This protease maintains activity at neutral pH and can liberate the chemokine domain of the neuronal chemokine Fractalkine (FKN), which feeds back onto microglia through the engagement of the CX3CR1 receptor (Clark et al., 2007, 2009). Here we review the contribution of spinal FKN/CX3CR1 signaling to neuro-immune interactions during neuropathic pain.

## The FKN/CX3CR1 signaling pair

Chemokines generally have a promiscuous relationship with their G-protein coupled receptors, with one chemokine binding to several different receptors and one receptor binding a range of ligands. However, the chemokine system is not functionally redundant (Schall and Proudfoot, 2011). One chemokine interaction, between FKN (CX3CL1) and its receptor CX3CR1, is a monogamous relationship. In addition, FKN is structurally unique amongst the family of chemokines; it is the only member of the CX3C family of chemokines and was first described as a potent attractant of immune cells (Bazan et al., 1997; Pan et al., 1997). The protein can exist in two forms, each of which mediates distinct biological actions: a membrane tethered protein and soluble forms containing the chemokine domain (Bazan et al., 1997).

FKN is expressed in both the periphery and the CNS. Pan et al. originally described FKN gene expression to be most abundant in the brain and heart, but absent from peripheral blood leukocytes (Pan et al., 1997). Endothelial and epithelial cells are the predominant FKN-expressing cells in the periphery. Indeed, FKN has been localized to endothelial cells of the skin (Papadopoulos et al., 1999, 2000), heart (Harrison et al., 1999), and lung (Foussat et al., 2000), and to intestinal epithelial and endothelial cells (Muehlhoefer et al., 2000). This constitutive expression of FKN is regulated by inflammatory stimuli; it is enhanced following exposure of these cells to Lipopolysaccharide (LPS; Pan et al., 1997), pro-inflammatory cytokines (Bazan et al., 1997; Muehlhoefer et al., 2000), and during inflammatory conditions such as Crohn’s disease (Muehlhoefer et al., 2000).

Neurons are the principle FKN expressing cells of the CNS, with endothelial cells in the brain showing little or no expression (Harrison et al., 1998; Nishiyori et al., 1998; Maciejewski et al., 1999; Hughes et al., 2002; Tarozzo et al., 2002, 2003). Likewise in the spinal cord FKN expression is restricted to neurons (Verge et al., 2004; Lindia et al., 2005; Clark et al., 2009; Yang et al., 2012). FKN expression has also been observed in the cell bodies of peripheral sensory neurons in the dorsal root ganglia (DRG; Verge et al., 2004), and in the central terminals of these neurons in the spinal dorsal horn in some studies (Verge et al., 2004; Yang et al., 2012), but not in others (Lindia et al., 2005; Clark et al., 2009). The expression profile of FKN has been confirmed by the recent development of a FKN reporter mouse (Kim et al., 2011). Peripherally, the expression of FKN in these mice is completely restricted to non-hematopoietic cells, with FKN-mCherry found in lung and intestinal epithelial cells and in kidney endothelial cells (Kim et al., 2011). Centrally, the steady-state neuronal location of FKN in some brain areas (hippocampus, striatum and cortical layer II) and spinal cord was also confirmed. However, FKN-mCherry expression was absent from the brainstem, midbrain, and cerebellum. FKN-mCherry was also not found in DRG cells (Kim et al., 2011), somehow questioning sensory neurons as a source of FKN outside the CNS under homeostatic conditions.

The shedding of membrane bound FKN into soluble forms represents a key regulatory mechanism for FKN signaling. The liberation of soluble FKN (sFKN) from endothelial and epithelial cells occurs both constitutively and in an inducible manner. In the context of vascular immune function, endothelial membrane bound FKN serves as an adhesion molecule, promoting the firm adhesion of leukocytes without the activation of integrins (Fong et al., 1998), whilst sFKN is a potent chemoattractant for monocytes, NK cells, T cells and B cells (Imai et al., 1997; Corcione et al., 2009). FKN/CX3CR1 interactions are also vital for many homeostatic processes, including the survival of CX3CR1^high^ blood monocytes (Landsman et al., 2009), wound healing (Ishida et al., 2008) and trans-endothelial migration for immune surveillance (Auffray et al., 2007). Constitutive shedding of membrane bound FKN is principally dependent on the metalloprotease ADAM-10 (a disintegrin and metalloprotease domain-10) (Hundhausen et al., 2003, 2007). Following stimulation of FKN-expressing cells with phorbol esters (e.g., Phorbol 12-myristate 13-acetate) shedding of mature FKN (∼100 kDa) into soluble FKN (∼80 kDa) is markedly enhanced; this inducible shedding is largely ADAM-17 (also known as TACE, tumor necrosis factor-α converting enzyme) dependent (Garton et al., 2001; Tsou et al., 2001). However, not all shedding of FKN observed can be accounted for by cleavage of ADAM-10 and ADAM-17, as following metalloproteinase inhibition some formation of sFKN is still observed (Hundhausen et al., 2003). Recent evidence indicates that the cysteine protease CatS expressed by vascular smooth cells also generates sFKN, although of a smaller size (∼50 kDa) (Fonovićc et al., 2013) than the sFKN liberated by the ADAMs. Indeed, in the spinal cord during chronic pain sFKN is liberated following cleavage of neuronal membrane bound FKN by CatS released by microglia (Clark et al., 2007, 2009). The possibility that ADAM-17 and/or ADAM-10 contributes to sFKN shedding in the spinal cord has not been evaluated, however FKN expression is absent from CNS endothelium (Harrison et al., 1998; Nishiyori et al., 1998; Maciejewski et al., 1999; Hughes et al., 2002; Tarozzo et al., 2002, 2003), therefore ADAM mediated cleavage of FKN in the CNS seems unlikely. Interestingly, different proteases may cleave FKN at diverse locations and it is likely that sFKN exists in several forms. ADAM-10 and ADAM-17 cleave FKN at different sites close to the plasma membrane (Bazan et al., 1997; Garton et al., 2001; Tsou et al., 2001), whilst the exact cleavage site of CatS has not yet been determined.

The CX3CR1 receptor was identified in humans (Imai et al., 1997; Combadiere et al., 1998) and rat (Harrison et al., 1994) in the 1990’s. Like all of the chemokine receptors, CX3CR1 is seven-transmembrane domained G-protein coupled receptor. CX3CR1 expression is abundant in both peripheral blood leukocytes and microglia in the CNS. The development of a transgenic mouse by Jung et al. in which the CX3CR1 gene was mutated to contain a green fluorescent protein (GFP) reporter gene (Jung et al., 2000), has allowed the pattern of CX3CR1 expression in the mouse to be analyzed in depth. Murine blood contains populations of monocytes (CD11b^+^ Gr1^low^) and Natural Killer cells that express CX3CR1. On the other hand, murine B-lymphocytes and T-lymphocytes (both resting and active), eosinophils and neutrophils are CX3CR1 negative. Expression of CX3CR1 is also found on both myeloid and lymphoid dendritic cells and populations of cutaneous Langerhans cells (Jung et al., 2000). It should be noted that the expression of CX3CR1 in human blood differs from that in the mouse, with expression observed in populations of human T-lymphocytes (Raport et al., 1995; Foussat et al., 2000). In the CNS, CX3CR1 is exclusively expressed by microglia. In both the mouse and the rat microglia in the brain express CX3CR1, with expression completely absent from astrocytes, oligodendrocytes and neurons (Harrison et al., 1998; Nishiyori et al., 1998; Jung et al., 2000). Likewise in the spinal cord CX3CR1 is exclusively expressed by microglial cells (Verge et al., 2004; Lindia et al., 2005; Zhuang et al., 2007; Yang et al., 2012; Clark et al., 2013). Controversial *in vitro* evidence for neuronal CX3CR1 expression in cultured hippocampal neurons (Meucci et al., 2000; Limatola et al., 2005), has not been confirmed *in vivo* using the CX3CR1-GFP reporter mouse (Jung et al., 2000), suggesting that such expression may be a phenomenon of the culture system. Critically the neuroprotective effects of FKN in hippocampal cultures originally attributed to a direct action on the hippocampal neurons themselves (Meucci et al., 2000), has been demonstrated to be mediated by microglial released mediators, and can be attributed to microglial contamination in the neuronal cultures (Lauro et al., 2008). Overall evidence indicates that in the CNS the FKN/CX3CR1 signaling pair are ideally located to mediate neuron-microglial communication, both during homeostatic and pathological processes.

In the brain FKN/CX3CR1 interactions are thought to play a homeostatic role in the regulation of microglia cell activity, contributing to the maintenance of a surveillance state in these cells. It has been demonstrated that FKN/CX3CR1 regulate hippocampal neurogenesis, synaptic pruning, synaptic plasticity, and are neuroprotective in a number of pathological conditions (Recently reviewed in Sheridan and Murphy, 2013). The role of FKN/CX3CR1 interactions in spinal homeostatic mechanisms remains to be determined. However, it has become evident that aberrant FKN/CX3CR1 signaling can contribute significantly to the pathogenesis of a number of chronic diseases (Nishimura et al., 2009; Jones et al., 2010; Clark et al., 2011; Liu and Jiang, 2011), perhaps unsurprising given the role of this pair in immune and inflammatory processes. Among these conditions, there is now extensive evidence to support a role for FKN/CX3CR1 signaling in the chronicity of pain.

## Spinal FKN/CX3CR1 and neuron-microglia communication during neuropathic pain

The first synapse in the nociceptive pathway, between the central terminals of primary afferent fibers and dorsal horn neurons in the spinal cord, is a key site at which modulation of nociceptive transmission can occur. Neuropathic pain is commonly modeled in rodents using surgical injury to a peripheral nerve, usually the sciatic nerve or a branch thereof, which induces robust and reproducible pain behaviors in the effected hind-paw. It is now well established that damage to a peripheral nerve causes disruption of homeostasis; as a result microglia (and astrocytes) in the vicinity of injured primary afferent terminals in the dorsal horn transition into pain-related enhanced response states (McMahon and Malcangio, 2009). Thus augmentation of neuron-microglia communication critically contributes to amplification of nociceptive transmission which occurs during neuropathic pain. In the dorsal horn, neuronal FKN and microglial CX3CR1 are ideally located to mediate neuron-microglia communication.

FKN in its soluble form is pro-nociceptive; intrathecal administration of the FKN chemokine domain (Milligan et al., 2004, 2005; Clark et al., 2007; Zhuang et al., 2007; Clark and Malcangio, 2012), but not full length FKN (Clark and Malcangio, 2012), induces hypersensitivity to both thermal and mechanical stimuli, which is entirely mediated via CX3CR1 (Milligan et al., 2004, 2005; Clark et al., 2007; Staniland et al., 2010). FKN induces nociceptive behaviors following activation of CX3CR1 and intracellular phosphorylation of microglial p38 Mitogen-activated protein kinase (MAPK; Clark et al., 2007; Zhuang et al., 2007) which subsequently stimulates release of pro-inflammatory mediators including Interleukin- 1β, Interleukin-6 and Nitric Oxide (Milligan et al., 2005).

Impairment of spinal FKN/CX3CR1 signaling represents a potential therapeutic avenue during chronic pain. Following injury to a peripheral nerve extensive upregulation of CX3CR1 occurs in spinal microglia (Verge et al., 2004; Lindia et al., 2005; Zhuang et al., 2007; Staniland et al., 2010), with FKN becoming *de novo* expressed in astrocytes in the spinal nerve transection model of peripheral nerve injury (Lindia et al., 2005), but not in other models (Verge et al., 2004; Zhuang et al., 2007; Staniland et al., 2010). Although levels of total FKN protein in the spinal cord remain unchanged following nerve injury (Verge et al., 2004; Lindia et al., 2005; Clark et al., 2009), sFKN levels in CSF are significantly elevated (Clark et al., 2009); thus there is enhanced availability of sFKN alongside enhanced CX3CR1 expression during neuropathic pain. In a number of models of peripheral nerve injury intrathecal administration of FKN or CX3CR1 neutralizing antibodies is able to attenuate neuropathic pain behaviors (Milligan et al., 2004; Clark et al., 2007; Zhuang et al., 2007); this is due to a reduced pro-nociceptive activity state of spinal microglia, as demonstrated by reduced p38 MAPK phosphorylation (Zhuang et al., 2007). The same effect is true for the development of bone cancer pain; the development of pain in animals with experimental bone cancer occurs concurrently with microgliosis and an increase in the expression of microglial CX3CR1 and p-p38. The onset of this pain can be significantly delayed by the intrathecal administration of a CX3CR1 neutralizing antibody (Yin et al., 2010; Hu et al., 2012) despite a lack of efficacy in suppressing bone pathology (Yin et al., 2010). Whilst neutralizing antibodies and modified FKN proteins have been utilized for proof of concept preclinical studies, the first CX3CR1 antagonist to show anti-inflammatory activity at both mouse and human CX3CR1 was recently described (White et al., 2010; Karlström et al., 2013).

**Figure 1:**
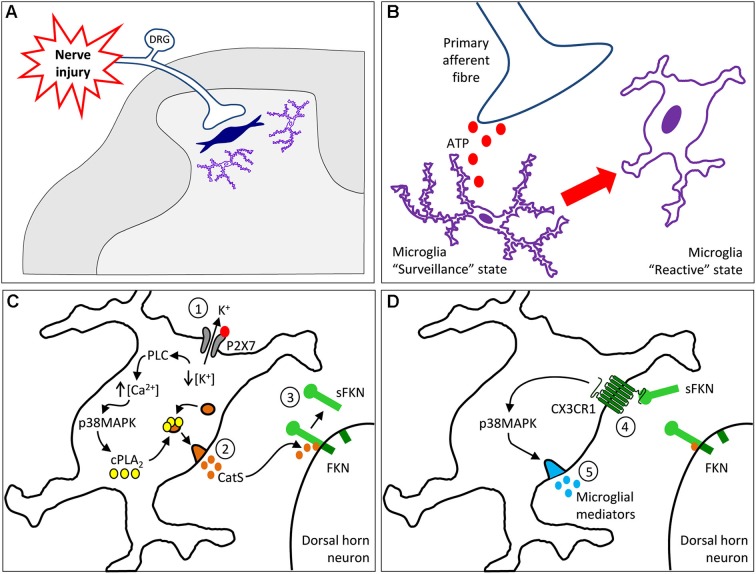
**Schematic illustrating the pro-nociceptive mechanism of CatS/FKN signaling in the spinal dorsal horn during neuropathic pain. (A–B)** In the dorsal horn area innervated by damaged fibers (**Panel A**) microglia transform from a surveillance state into a reactive state following exposure to injury induced factors released by primary afferent terminals, including Adenosine tri-phosphate (ATP; **Panel B**). **(C)** High concentrations of extracellular ATP leads to P2X7 receptor activation on microglia (1), which ultimately leads to the release of CatS. A decrease in intracellular potassium concentration following efflux through the P2X7 receptor activates phospholipase C (PLC), resulting in an increase in intracellular calcium and phosphorylation of p38 MAPK. P38 phosphorylation then allows phospholipase A_2_ (PLA_2_) mediated translocation of CatS containing lysosomes to the cell membrane, whereby exocytosis releases CatS into the extracellular space (2). Extracellular CatS is then able to cleave membrane bound FKN from dorsal horn neurons, liberating soluble FKN (sFKN) (3). **(D)** sFKN feeds back onto the microglial cells via the CX3CR1 receptor (4) to further activate the p38 MAPK pathway and release inflammatory mediators, (5) that activate neurons and result in chronic pain. Abbreviations: DRG, dorsal root ganglia, cPLA_2_, cytosolic PLA_2_.

Critically, we demonstrated that CX3CR1 deficient mice show deficits in neuropathic pain; these mice do not develop mechanical allodynia, and have reduced hypersensitivity to thermal stimuli, following peripheral nerve injury, compared to wild-type mice (Staniland et al., 2010). The deficits in the development of neuropathic pain behaviors correlate with a reduction in microglial cell activity in these mice, as spinal microglial response is milder in knockout mice. Interestingly, extensive infiltration of macrophages occurs at the site of nerve injury; however no difference in the number of infiltration macrophages was identified between genotypes (Staniland et al., 2010), suggesting that CX3CR1 expressing macrophages in the nerve contribute little to neuropathic pain in this model. In the spinal cord the pro-nociceptive actions of sFKN are mediated following its liberation by the lysosomal protease CatS (Recently reviewed in Clark and Malcangio, 2012). Following peripheral nerve injury CatS is upregulated in microglial cells in the area innervated by damaged primary afferent terminals (Clark et al., 2007). CatS is released from microglia in a P2X7 dependent manner (Clark et al., 2010), cleaving FKN located on the cell membrane of dorsal horn neurons to liberate the soluble chemokine domain of FKN, which then signals to microglia via CX3CR1 (Clark et al., 2007) (as summarized in Figure 1). Following peripheral nerve injury significant levels of sFKN can be detected in the CSF, along with enhanced CatS activity (Clark et al., 2009). FKN cleavage in the dorsal horn occurs under highly regulated conditions associated with increased nociception (Clark et al., 2009). In neuropathic spinal cord slices electrical stimulation of injured dorsal roots induces liberation of sFKN (Clark et al., 2009). The liberation of sFKN is only associated with conditions in which microglia are in an reactive state, for example following nerve injury or stimulation with LPS, and is completely dependent on CatS activity (Clark et al., 2009). Indeed, impairment of FKN signaling, either by neutralization of spinal FKN or by knock-out of CX3CR1, is able to completely prevent the pro-nociceptive effects of intrathecal CatS (Clark et al., 2007).

The pro-nociceptive effects of the CatS/FKN/CX3CR1 signaling are critical for the maintenance phase of neuropathic pain. Both intrathecal (Clark et al., 2007) and systemic (Barclay et al., 2007; Irie et al., 2008; Zhang et al., 2014) delivery of CatS inhibitors reverse established pain behaviors following peripheral nerve injury to varying degrees. We have shown that CatS inhibitors are ineffective when given intrathecally during the initiation phase of neuropathic pain (at day 3 post-injury) (Clark et al., 2007) when expression levels are low both peripherally (Barclay et al., 2007) and in the spinal cord (Clark et al., 2007), but effectively reverse established pain behavior when delivered intrathecally at later timepoints when expression of CatS is high (Clark et al., 2007). Indeed, a recent study has confirmed our findings, demonstrating that when administered systemically an inhibitor of CatS reverses neuropathic pain behaviors commencing on day 5 post-injury, but is ineffective when delivered between day 0 and 4 (Zhang et al., 2014). In addition, CatS null mice develop pain behavior that is equivalent to wild-type mice immediately following nerve injury, only demonstrating a reduction in allodynia compared to wild-types from day 3 post-injury onwards (Zhang et al., 2014).

In summary, following peripheral nerve injury disruption of homeostasis leads to microglia-driven aberrant FKN/CX3CR1 signaling in the dorsal horn of the spinal cord which maintains maladaptive neuron-microglia signaling and critically contributes to the chronicity of neuropathic pain.

## Conclusions

A greater understanding of the nature of neuron-microglia interactions during neuropathic pain states has led to the identification of new microglial therapeutic targets, including chemokine receptors such as CX3CR1 and the lysosomal protease CatS (Clark et al., 2011; Clark and Malcangio, 2012). Intracellular signaling pathways, most prominently p38 MAPK phosphorylation, mediate the release of pro-nociceptive mediators by spinal microglial cells comprising cytokines and proteases. Accordingly, the inhibition of microglial targets including CX3CR1, p38 MAPK and CatS can attenuate mechanical hypersensitivity in chronic pain models. Importantly, a CNS penetrant p38 MAPK inhibitor has demonstrated initial success in neuropathic pain patients (Anand et al., 2011) suggesting that impedance of microglial targets is a promising therapeutic avenue.

## Conflict of interest statement

The authors declare that the research was conducted in the absence of any commercial or financial relationships that could be construed as a potential conflict of interest.
